# Familial Psychosis Associated With a Missense Mutation at *MACF1* Gene Combined With the Rare Duplications DUP3p26.3 and DUP16q23.3, Affecting the *CNTN6* and *CDH13* Genes

**DOI:** 10.3389/fgene.2021.622886

**Published:** 2021-04-07

**Authors:** Josep Pol-Fuster, Francesca Cañellas, Laura Ruiz-Guerra, Aina Medina-Dols, Bàrbara Bisbal-Carrió, Víctor Asensio, Bernat Ortega-Vila, Diego Marzese, Carme Vidal, Carmen Santos, Jerònia Lladó, Gabriel Olmos, Damià Heine-Suñer, Konstantin Strauch, Antònia Flaquer, Cristòfol Vives-Bauzà

**Affiliations:** ^1^Health Research Institute of Balearic Islands (IdISBa), Palma, Spain; ^2^Department of Biology, University of Balearic Islands (UIB) and Institut Universitari d’Investigacions en Ciències de la Salut, IUNICS, Palma, Spain; ^3^Psychiatry Service, University Hospital Son Espases (HUSE), Palma, Spain; ^4^Research Unit, HUSE, Palma, Spain; ^5^Genomic Service Balearic Islands (GEN-IB), HUSE, Palma, Spain; ^6^Chair of Genetic Epidemiology, IBE, Faculty of Medicine, LMU Munich, Munich, Germany; ^7^Institute of Genetic Epidemiology, Helmholtz Zentrum München-German Research Center for Environmental Health, Neuherberg, Germany

**Keywords:** copy number variant, whole exome sequencing, schizophrenia, *CDH13*, *CNTN6*, *MACF1*

## Abstract

Psychosis is a highly heritable and heterogeneous psychiatric condition. Its genetic architecture is thought to be the result of the joint effect of common and rare variants. Families with high prevalence are an interesting approach to shed light on the rare variant’s contribution without the need of collecting large cohorts. To unravel the genomic architecture of a family enriched for psychosis, with four affected individuals, we applied a system genomic approach based on karyotyping, genotyping by whole-exome sequencing to search for rare single nucleotide variants (SNVs) and SNP array to search for copy-number variants (CNVs). We identified a rare non-synonymous variant, g.39914279 C > G, in the *MACF1* gene, segregating with psychosis. Rare variants in the *MACF1* gene have been previously detected in SCZ patients. Besides, two rare CNVs, DUP3p26.3 and DUP16q23.3, were also identified in the family affecting relevant genes (*CNTN6* and *CDH13*, respectively). We hypothesize that the co-segregation of these duplications with the rare variant g.39914279 C > G of *MACF1* gene precipitated with schizophrenia and schizoaffective disorder.

## Introduction

Schizophrenia (SCZ), despite a relatively low prevalence, estimated to be around 0.75% (Moreno-Küstner et al., 2018), is ranked among the top 15 leading causes of disability worldwide (GBD 2016 Disease and Injury Incidence and Prevalence Collaborators, 2017). As much as 80% (Lichtenstein et al., 2009; Hilker et al., 2018) of SCZ risk may be explained by genetic factors, including both common and rare genomic variants. Through genome-wide association studies (GWAS) hundreds of variants have been identified (Manolio et al., 2009; Schizophrenia Working Group of the Psychiatric Genomics Consortium, 2014; Bipolar Disorder and Schizophrenia Working Group of the Psychiatric Genomics Consortium, 2018), with an overall contribution of around 25% (Lee et al., 2012). Common SNPs have shown weak individual effects (odds ratios, <1.2); however, copy number variants (CNVs), with a high impact on gene dosage, have been implicated in SCZ with larger effect size compared to SNPs (odds ratios, 2–57) (Rees et al., 2014). Moreover, increased SCZ susceptibility has been associated with increased frequencies of CNVs (The International Schizophrenia Consortium, 2008; Grozeva et al., 2012; Szatkiewicz et al., 2020) and large deletions (Bergen et al., 2012; Szatkiewicz et al., 2020). In addition to CNVs, rare *de novo* or recent single-nucleotide variants (SNVs) with large effects have emerged as highly penetrant contributors (Purcell et al., 2014; Genovese et al., 2016; Singh et al., 2016; Steinberg et al., 2017), thanks to the availability of next-generation whole genome or exome sequencing. Overall, psychotic disorders as SCZ or bipolar disorder (BD) are the consequence of inheriting a genomic architecture where rare variants with high impact precipitate together with common variants with lower impact. Family-based studies are a powerful strategy to identify rare SNVs or CNVs without the need of collecting large cohorts. The purpose of the current study was to conduct a systematic genomic approach to study a Majorcan family enriched for psychosis, having two subjects with SCZ, and two with schizoaffective disorder (SCA).

## Materials and Methods

### Psychiatric and Cognitive Assessments

All family members underwent semi-structured interviews, using the Spanish version of the Structured Clinical Interview for Diagnostic and Statistical Manual of Mental Disorders (DSM-IV) Axis I (SCID-I), the Positive and Negative Syndrome Scale (PANNS), and the Diagnostic Interview for Genetic Studies (DIGS) (Supplementary Table 1). The cognitive assessment was performed using the Measurement and Treatment Research to Improve Cognition in Schizophrenia Consensus Cognitive Battery (MATRICS). It includes the assessment of 7 cognitive domains: speed of processing, attention/vigilance, working memory, verbal learning, visual learning, reasoning and problem solving, and social cognition. The same trained psychologist performed all cognitive assessments and most of them were done domiciliary. Medication status was taken into account at the moment of the assessment.

### Subject Description

**FIGURE 1:**
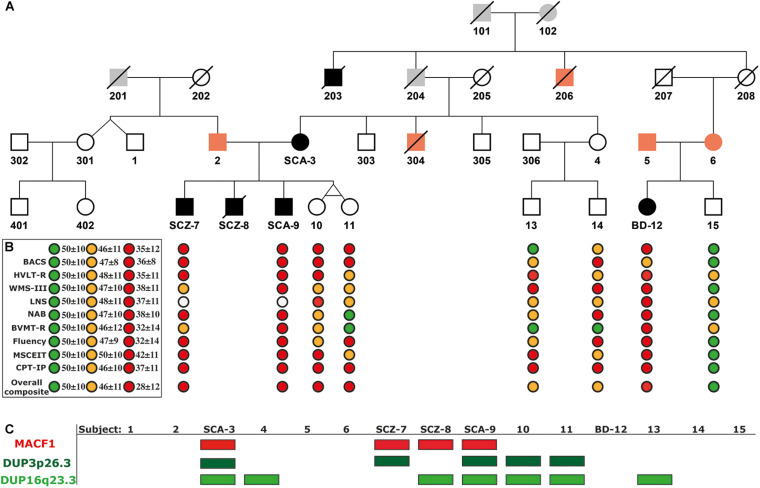
Extended Pedigree of the Majorcan Family with the cognitive evaluation and the segregation of genomic variants associated with psychosis. **(A)** Pedigree. In black, subjects diagnosed with psychosis; in orange, subjects with a history of mental disorders without psychosis; in gray, unknown individuals. **(B)** Cognitive assessment. TMT, Trail Making Test (Part A); BACS, Brief Assessment of Cognition in Schizophrenia (Symbol coding); HVLT-R, Hopkins Verbal Learning Test-Revised; WMS-III, Wechsler Memory Scale-Third Edition (Spatial Span); LNS, Letter-Number Span Test; NAB Mazes, Neuropsychological Assessment Battery Mazes; BVMT-R, Brief Visuospatial Memory Test-Revised; Fluency: Category Fluency Test, Animal naming; MSCEIT, Mayer-Salovey-Caruso Emotional Intelligence Test; CPT-IP, Continuous Performance Test-Identical Pairs; Overall Composite Score, Cognitive index obtained summing all the tests scores and standardizing the result to a T score. In green, values of healthy controls; in orange, values of unaffected relatives with scores below the 25th percentile within their category; in red, values of patients with psychosis. **(C)** Segregation of the c.39,914,279 C > G mutation in the *MACF1* gene and the duplications DUP3p26.3 and DUP16q23.3.

The proband of the Majorcan Family affected by SCA is a mother of 5 children (subject SCA-3, Figure 1A). She is characterized by alternating depressive and manic episodes accompanied by delusional ideation of paranoid type. The onset of symptoms was after the birth of her twin daughters. The proband has three sons (Figure 1A): SCZ-7 presents SCZ from the age of 19 with predominant negative symptoms. SCZ-8 onset with psychotic symptoms when he was 16 years old; he was diagnosed with paranoid SCZ and died by suicide in 2014. And SCA-9 started to evidence behavioral disorders, anxiety and somatic symptoms at the age of 14, diagnosed later as SCA. The twin daughters do not present psychiatric symptoms (subjects 10 and 11) at the age of 33 years old. The family history of the proband included a brother who died of cirrhosis due to alcoholism (subject 304, Figure 1A), an uncle with autism and psychosis (subject 203, Figure 1A), another uncle with mental illness, and epilepsy (subject 206, Figure 1A), and a cousin with anxiety (subject 6, Figure 1A). Subject 6 has a daughter affected by BD I (subject BD-12, Figure 1A). Her father (subject 5, Figure 1A) is diagnosed with major depressive disorder (MDD). The analysis presented in this article is focused on the nuclear family comprised of SCA-3, her husband, subject 2, and their children, SCZ-7, SCZ-8, SCA-9, and the twins, 10 and 11 (Figure 1A). Additionally, some extended family members of this nuclear family could be analyzed and were checked for the variants identified (Subjects 1, 4, 5, 6, 13, 14, BD-12, and 15; Figure 1). All studied family members signed the informed consent and the project was approved by the ethics committee of the Balearic Islands (CEI-IB).

### Blood Collection and DNA Extraction

Peripheral blood samples were obtained in EDTA tubes for the 7 members of the nuclear family and 8 additional subjects of the extended family. DNA extraction was performed using the DNA Blood Extraction Kit (5Prime^®^) according to the manufacturer’s protocol.

### Genotyping by SNP Array

Genotyping was carried in the Genome Analysis Centre (GAC, Helmholtz Zentrum München) using the Infinium Global Screening Array-24 v1.0 (GSA) from Illumina (Illumina Inc., San Diego, CA, United States), which includes 642,824 SNPs. Besides, a pool of 57,254 SNPs previously related to neurological disorders was also genotyped. The genotype calling and CNV analysis were performed using the Genome Studio 2.0 (Illumina Inc. San Diego, CA, United States). Family samples genotyped by SNP array included all the samples of the nuclear family (subjects 2, SCA-3, SCZ-7, SCZ-8, SCA-9, 10, and 11), and extended family members (subjects 1, 4, 5, 6, 13, 14, BD-12 and 15). In total, 15 samples were genotyped by SNP array (Figure 1). For the CNV analysis, the *cnv*Partition algorithm, implemented in Genome Studio 2.0, was used (Illumina Inc. San Diego, CA, United States), taking as a reference GRCh37/hg19. This algorithm is based on two parameters: the B allele frequency (BAF) and the LogRRatio (LRR); both can be used to test the genotyping quality of the samples and to check the presence of CNVs across the genome. The BAF is a measure of allelic imbalance. In a normal well-genotyped sample, three genotypes are expected: homozygous AA, heterozygous AB, and homozygous BB. Once referred to the B allele, BAF is expected to have three discrete values: 0, 0.5, and 1 (representing AA, AB, and BB genotypes, respectively). R is defined as the sum of the probe intensities used to genotype the different markers. When R is normalized, it becomes the LRR which is a measure of relative intensity, the logarithm (base 2) of the observed value of R (observed probe intensity) divided by the expected value (expected probe intensity) (Peiffer et al., 2006). Before running CNV analysis, array samples were quality controlled considering the missing call rate per subject, the number of null SNPs of a sample over the total number of genotyped SNPs. The missing call rate for all genotyped samples was below 1% (call rate over 99%) except for sample SCZ-9 which had a 10.92% missing call rate (call rate 89%). When the SCZ-9 array sample was visualized in Genome Studio 2.0, it could be observed that both the BAF and the LRR were altered for this sample. These results were consistent with a pattern of contamination, reason why this sample was excluded from the analysis. CNVs < 80,000bp were filtered. CNVs were visually inspected using Genome Studio 2.0, and CNVs carried by more than one subject of the nuclear family were validated by Digital Droplet PCR (ddPCR) (Supplementary Table 2).

### CNV Analysis by ddPCR

To confirm the CNVs identified by SNP array, Droplet Digital PCR (ddPCR) was performed. Before ddPCR, the genomic DNA of each family member was digested for 1 h at 37°C with the restriction enzyme Hind III. ddPCR was performed using the following set of primers: for DUP16q23.3 5′-TTGGTGTTTGACCCTGTGAA-3′ (Forward) and 5′-TGA GCTAGGGCTCCCACTTA-3′ (Reverse); for DUP3p26.3 5′-TCAGTGAAGTGCCTGGTTTG-3′ (Forward) and 5′-GGCT GTTCCATGAGGAATGT-3′) (Reverse). And the following 5′-FAM probes with a 3′-BHQ1 quencher: for DUP16q23.3 5′-FAM-TTTGGATTGCTTTGCCTACC-BHQ1-3′, and DUP3p 26.3 5′-FAM-CTAGGCTGGGCTCACTTGTC-BHQ1-3′. RPP30 was used as a reference control: 5′GATTTGGACCTG CGAGCG-3′ (Forward), 5′-GCGGCTGTCTCCACAAGT-3′ (Reverse), and the probe VIC-CTGACCTGAAGGCTCT-BHQ1 (Supplementary Table 3). The PCR was performed in a C1000 touch thermocycler (Bio-Rad, Hercules, CA, United States) with the following protocol: Initial denaturalization at 95°C for 10 min, followed by 39 cycles at 94°C for 30 s and an extension at 57°C for 1 min, with a final denaturing at 98°C for 10 min. Droplet analysis was performed using the Q200 Droplet Reader (Bio-Rad). CNVs were calculated using the QuantaSoft software (Bio-Rad)^1^.

### Whole Exome Sequencing (WES)

WES included three affected members (SCA-3, SCZ7, and SCZ-8) and one of the unaffected twin sisters (subject 10). Additionally, subject BD-12 from the extended family was also whole-exome sequenced. The SureSelectXT Human All Exon V6 capture library from Agilent Technologies (Santa Clara, CA, United States) was used for target enrichment. Exome sequencing was performed on an Illumina HiSeq4000 sequencer (Illumina Inc. San Diego, CA, United States), using 100 base paired-end sequencing, by BGI Tech Solutions. The workflow to obtain variant call format (VCF) files from raw data (FASTQ) provided by Macrogen was based on GATK Best Practices (Depristo et al., 2011). FASTQ files, containing raw unmapped reads and Phred scores, were quality controlled using the FastQC v0.11.2^2^. Low-quality sequences (Phred score < 20) and adaptors were removed using Cutadapt (v1.4)^3^. QC sequences were aligned against the reference human genome (GRCh37/hg19) using the BWA-MEM algorithm implemented in the Burrows-Wheeler Alignment tool (BWA v0.7.12)^4^. Aligned data in SAM (Sequence Alignment/Map) format were then sorted and converted into BAM files using SAMtools (v1.1)^5^. To generate new BAM files, PCR duplicates were removed using Picard Tools v1.118^6^, and realignment around INDELS and base recalibration was performed (BQRS) using Genome Analysis Toolkit (GATK). SNV and INDEL calling was carried from the cleaned BAM files using GATK (v3.3.0)^7^ producing unfiltered primary VCF files; which were then hard filtered to generate the definitive VCF files. To verify the exome sequencing results, Sanger sequencing was performed in the Genetic Analyzer AbiPrism 3700 (Applied Biosystems, Foster City, CA, United States) using standard polymerase chain reaction conditions. Results were visualized using Sequencing Analysis 5.1.1. (Applied Biosystems).

### Analysis of Rare SNV and CNV Variants

VCF files were annotated using variant annotation and effect prediction tool (SnpEff and SnpSift; Version 4.3)^8^ (Cingolani et al., 2012). It allows annotating variants using the dbNSFP database, an integrated database of functional predictions from multiple algorithms. Variants were annotated, based on predictions comprised in the 2.1 version of the dbNSFP database, for protein impact using SIFT (Kumar et al., 2009), PolyPhen2 (Carter et al., 2013), LRT (Chun and Fay, 2009), MutationAssessor (Reva et al., 2007), Meta SVM and LR (Dong et al., 2015), VEST3 (Carter et al., 2013), FATHMM (Shihab et al., 2013), and MutationTaster (Schwarz et al., 2010); and for conservation using SiPhy_29way_logOdds (Garber et al., 2009), PhyloP100way_vertebrate (Pollard et al., 2010), PhastCons100way_vertebrate (Felsenstein and Churchill, 1996) and GERP++ (Davydov et al., 2010). The resulting files were managed with R 3.6.1. Alternatively, VCF files were managed using ENLIS Genome Research version 1.9 (Berkeley, CA, United States). ENLIS uses its annotation pipeline. The RVI Score (Residual Variation Intolerance Score)^9^ (Petrovski et al., 2013) was also used to assess the tolerance to variation of the genes affected by a variant in the family. RVIS is a ranked score of genes according to the number of common functional genetic variants they carry compared to the genome-wide expectation. RVIS is based on 6,503 WES samples from the NHLBI Exome Sequencing Project and it is the result of regressing the number of common functional variants on the total number of protein-coding variants. The loss-of-function observed/expected upper bound fraction (LOEUF) (Karczewski et al., 2019) was also considered as an indicator of gene tolerability to predicted loss of function variants. It is based on the continuous metric of the observed/expected (o/e) ratio and its confidence interval^10^.

Shared variation among affected individuals was filtered for Read Depth >10 and Minor Allele Frequency (MAF) <0.01. All genomic data for molecular variants in this study were compatible with Genome build GRCh37. Allele frequencies were checked in 1000G^11^ (Birney and Soranzo, 2015) and in ExAC and its successor, the Genome Aggregation Database (gnomAD) (see text footnote 10) (Lek et al., 2016). Before validation, SNV variants were checked directly from BAM files using Integrative Genomics Viewer (IGV)^12^ (Thorvaldsdottir et al., 2013). The identified SNV variants and the genes affected by them were checked on VarElect^13^ (Stelzer et al., 2016), DisGeNET^14^ (Piñero et al., 2020), and Schizophrenia Exome Sequencing Genebook (Purcell et al., 2014) to identify potential previous reports of these variants or genes in psychosis. Regarding CNVs, we checked different databases to find previous reports of the CNVs identified in the family and to study their pathogenicity and conservation: DGV (Database of genomic variants)^15^ (MacDonald et al., 2014) and DECIPHER (DatabasE of genomiC varIation and Phenotype in Humans using Ensembl Resources)^16^ (Firth et al., 2009). VarSome^17^ (Kopanos et al., 2019) and UniProt^18^ (Bateman, 2019) were used to predict the levels of variant penetrance.

## Results

### The Majorcan Family Is Characterized by Poor Cognitive Profiles

Psychotic subjects obtained lower overall composite scores compared to healthy family controls (Supplementary Table 4). Once scores were compared with normal population values obtained from Mucci et al. (2017) (Figure 1B), all family members performed below average. Processing Speed was especially low in subjects SCZ-7 and SCA-9, obtaining both of them the lowest possible scores in TMT and BACS tests (percentiles <0.1 considering their reference group matched for age and sex; Supplementary Table 4). Yet, these subjects performed better in fluency evaluation. Their sisters (subjects 10 and 11) and subject BD-12 also had low scores in processing speed (percentiles <5 considering their reference group matched for age and sex; Supplementary Table 4). Subjects 13 and 14 had scores in the range of healthy individuals. Working memory was especially low in the affected subject BD-12. LNS was not tested on affected subjects SCZ-7 and SCA-9 because they could not remember the alphabet.

### Identification of Two Inherited CNVs Associated With Psychosis

**FIGURE 2:**
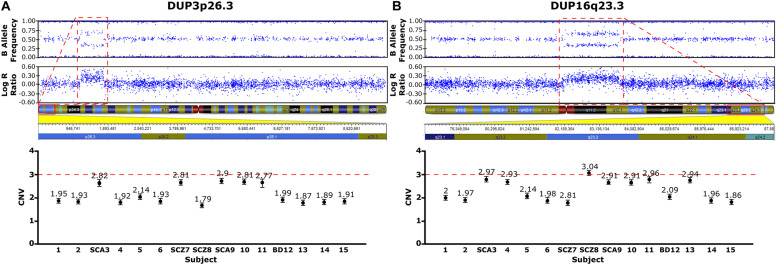
CNVs Identified in the Family Pedigree Associated with Psychosis. Upper panels, CNVs detected using the *cnv*Partition algorithm, based on the B allele frequency and the log R ratio (LRR: logarithm of the observed/expected probe intensity). Duplications identified (red rectangles), 3p26.3 **(A)** and DUP16q23.3 **(B)** are defined by allele frequencies of 0.33 and 0.66. The genetic map of the genomic regions containing the duplications are shown below with the affected genes highlighted in red. Lower panels, CNVs validated by ddPCR. Values close to 3 are indicative of a duplication **(A,B)**.

Two rare duplications (DUP3p26.3 and DUP16q23.3) were identified in the nuclear family (Figures 2A,B and Supplementary Table 2). These CNVs were validated by ddPCR in all studied family members (Figures 2A,B, lower panels). DUP3p26.3 is located on chromosome 3 (from 1,159,787 to 1,781,739 bp) and contains the contactin 6 (*CNTN6*) gene. Within these coordinates, 48 CNVs of similar size (under 1 Mb), containing the *CNTN6* gene, have been reported (DECIPHER) (Firth et al., 2009). The gain of dose of this gene has been associated with intellectual disability (ID), delayed speech and language development, cognitive impairment, micro and macrocephaly, and autism (DECIPHER). The mother (SCA-3) carries DUP3p26.3 and transmitted it to her affected sons SCZ-7 and SCA-9 (Figure 2A). The healthy twin daughters also carry this duplication (subjects 10 and 11, Figure 2A). None of the extended relatives of the family carries it.

DUP16q23.3 is located on chromosome 16 (from 82,180,075 to 83,664,582 bp) and contains the cadherin-13 (*CDH13*) gene. 28 CNVs of similar size (under 2 Mb) have been reported within these coordinates, 18 of which are duplications affecting the *CDH13* gene and are associated with ID, delayed speech, language development, and global developmental delay (DECIPHER). The mother (SCA-3) carries this duplication and she transmitted it to her affected sons SCZ-8 and SCA-9 (Figure 2B). Interestingly, the healthy twin daughters also carry this duplication (subjects 10 and 11, Figure 2B). Among the extended relatives of the family, DUP16q23.3 is also carried by an unaffected sister of the mother (subject 4), who transmitted it to one of her two unaffected sons, subject 13 (Figure 2B and Supplementary Table 2).

### Contributions of Rare SNVs Identified by WES

To identify rare variants of potentially higher penetrance, WES analysis was restricted to MAF < 1% according to ExAC, 1000G, and gnomAD. The psychotic patients shared 19 rare missense SNVs in conserved regions according to different conservation algorithms that were absent from the healthy twins (Tables 1, 2). Nonsense variants were not identified (Tables 1, 2). Regarding the 19 shared missense variants, all except two (rs146468598 and rs112913396) affect genes that are expressed in the cerebral cortex (GTEx database; Table 1). Out of these 17 variants, 9 are predicted “VUS” and 8 “benign” or “likely benign” by VarSome (Table 1). Among the 9 variants predicted “VUS,” 3 variants stood out because they were predicted “deleterious” by almost all protein prediction algorithms; p. P-56-L in MTMR11, p. P-241-S in TINF2, and p. Y-161-C in ZRANB3 (Table 3). Nevertheless, to our knowledge, the genes affected by these variants have no described functions in the central nervous system and have not been associated with SCZ or any other mental disorder. Only three variants among the 17 expressed in the cerebral cortex affect genes previously associated with SCZ according to DisGeNET (Piñero et al., 2020): p. R-1543-H in GLI2, p. T-4642-S in MACF1, and p. R-39-Q in MCTP2 (Table 1). Out of these three, only the *MACF1* variant is predicted “VUS” (Table 1). Moreover, the expression of *MCTP2* [median Transcripts Per Million (TPM) below 1] and *GLI2* (median TPM below 1.5) is low in the cerebral cortex (GTEx database). The variant located in the *MACF1* gene is located in the most conserved region among the 19 variants identified (Table 2). The variant in *GLI2* is also located in a very conserved region while the variant located in *MCTP2* is located in a less conserved region (Table 2). Moreover, among the 19 genes affected by the variants shared by psychotic individuals, *MACF1* is the most intolerant to variation (RVIS = −3.92 (0.21%); LOEUF = 0.084; Supplementary Table 5). Protein prediction algorithms show mixed results for the variants in *MACF1* and *GLI2* while the variant in *MCTP2* is predicted to be benign by all protein prediction algorithms used (Table 3). Given these results, we decided to analyze the segregation of the variants in *MACF1* and *GLI2*. The mother (SCA-3) transmitted the g.39914279 C > G (p. T4642S) mutation in *MACF1* to her three affected sons (SCZ-7, SCZ-8, and SCA-9), but not to her healthy twin daughters (subjects 10 and 11) (Figure 1C). Besides, this variant was not detected in any of the extended family members that were analyzed. The variant g.121748118 G > A (p. R1543H) in the *GLI2* gene was transmitted by the mother to her affected sons SCZ-7 and SCZ-8. The other affected individual, SCA-9, did not carry this variant which was neither detected in any of the extended family members analyzed. The presence of the duplications DUP3p26.3 and DUP16q23.3 *per se* is not sufficient to precipitate with psychosis, since non-affected subjects carry both duplications (i.e., the twin sisters, subjects 10 and 11). The psychotic phenotype may have precipitated once patients inherited one of these two duplications together with the rare variant c.39,914,279 C > G in the *MACF1* gene.

**TABLE 1:** Rare non-synonymous variants identified in conserved regions by WES shared only among affected subjects.

Chr	bp	Ref	Alt	rs	Gene	Protein Impact	aa Change	GTEX	VarSome	DisGeNET	ExAC	gnomAD	1000G
1	39914279	C	G	rs376728302	*MACF1*	MISSENSE	T-4642-S	Yes	VUS	Yes	0.000058	0.000092	NA
1	149907574	G	A	rs202234090	*MTMR11*	MISSENSE	P-56-L	Yes	VUS		0.000115	0.000159	NA
2	136107663	T	C	rs183525970	*ZRANB3*	MISSENSE	Y-161-C	Yes (low)	VUS		0.001391	0.001672	0.001374
2	219528732	C	T	rs138321594	*RNF25*	MISSENSE	R-443-Q	Yes	VUS		0.000069	0.000089	NA
3	102187900	A	G	rs145497598	*ZPLD1*	MISSENSE	K-301-R	Yes (Very low)	VUS		0.000025	0.000021	NA
5	54721089	G	A	rs201703457	*PPAP2A*	MISSENSE	T-268-I	Yes	VUS		0.000016	0.000011	NA
5	175812257	G	A	rs35916027	*NOP16*	MISSENSE	R-120-C	Yes	VUS		0.004062	0.004068	0.005037
14	24709965	G	A	rs17102311	*TINF2*	MISSENSE	P-241-S	Yes	VUS		0.002158	0.002167	0.004121
22	38061637	A	T	rs142028345	*PDXP*	MISSENSE	Y-217-F	Yes	VUS		0.007042	0.000004	0.005952
1	23743859	T	C	rs192746462	*TCEA3*	MISSENSE	E-88-G	Yes (low)	Likely Benign		0.004760	0.004642	0.002289
2	109382936	T	A	rs61748150	*RANBP2*	MISSENSE	S-1981-T	Yes	Benign		0.008895	0.008229	0.006410
2	121748118	G	A	rs138987487	*GLI2*	MISSENSE	R-1543-H	Yes (low)	Benign	Yes	0.000502	0.000534	0.000916
2	230723775	G	A	NA	*TRIP12*	MISSENSE	S-205-L	Yes	Benign		0.000025	0.000028	NA
4	39450295	G	A	rs143809363	*KLB*	MISSENSE	V-1042-I	Yes (low)	Likely Benign		0.003534	0.003275	0.000458
9	79323853	C	T	rs200875180	*PRUNE2*	MISSENSE	D-1113-N	Yes	Likely Benign		0.003781	0.003776	0.000916
10	81925866	T	C	rs72807973	*ANXA11*	MISSENSE	I-278-V	Yes	Benign		0.002990	0.003189	0.003205
15	94841610	G	A	rs149237812	*MCTP2*	MISSENSE	R-39-Q	Yes (low)	Likely Benign	Yes	0.001400	0.001199	0.000916
2	238289767	T	C	rs112913396	*COL6A3*	MISSENSE	D-563-G		VUS		0.002314	0.002079	0.000916
5	56777915	C	T	rs146468598	*ACTBL2*	MISSENSE	R-207-Q		VUS		0.001820	0.002011	0.003205

*19 variants with a MAF < 1% were shared by the 3 psychotic patients.*

Chr, chromosome; bp, base pair; Ref, reference Allele; Alt, alternate allele; GTEX, GTEX expression; DisGeNET, previous relationship to SCZ; VUS, variant of uncertain significance.

*Low median tpm below 2, **Very low median tpm below 0.05, ***Genetic coordinates are given taking as a reference GRCh37/hg19, ****Variants were annotated based on the 2.1 version of the dbNSFP database, *****Allele frequencies from gnomAD were obtained from the 2.1.1 gnomAD version.

**TABLE 2:** Conservation algorithms.

Chr	bp	Ref	Alt	rs	Gene	SiPhy	GERP_RS	GERP_NR	phyloP100	phastCons100
5	56777915	C	T	rs146468598	*ACTBL2*	15.631	4.91	4.91	7.645	0.989
10	81925866	T	C	rs72807973	*ANXA11*	9.0566	1.76	5.6	3.668	0.879
2	238289767	T	C	rs112913396	*COL6A3*	15.793	5.6	5.6	6.22	1
2	121748118	G	A	rs138987487	*GLI2*	18.456	4.98	4.98	9.657	1
4	39450295	G	A	rs143809363	*KLB*	12.583	4.83	5.68	1.477	0.998
1	39914279	C	G	rs376728302	*MACF1*	20.393	5.95	5.95	7.818	1
15	94841610	G	A	rs149237812	*MCTP2*	9.3437	3.19	5.13	1.658	0.044
1	149907574	G	A	rs202234090	*MTMR11*	12.986	4.68	4.68	3.916	1
5	175812257	G	A	rs35916027	*NOP16*	11.072	3.46	6.17	3.367	0.423
22	38061637	A	T	rs142028345	*PDXP*	10.036	4.54	5.58	4.666	1
5	54721089	G	A	rs201703457	*PPAP2A*	14.027	4.8	5.66	5.819	1
9	79323853	C	T	rs200875180	*PRUNE2*	14.954	5.94	5.94	4.165	0.984
2	109382936	T	A	rs61748150	*RANBP2*	16.063	5.75	5.75	5.214	1
2	219528732	C	T	rs138321594	*RNF25*	8.8576	3.74	5.55	1.989	0.99
1	23743859	T	C	rs192746462	*TCEA3*	11.332	5.02	5.02	1.69	1
14	24709965	G	A	rs17102311	*TINF2*	8.383	3.46	4.36	1.43	0.942
2	230723775	G	A	NA	*TRIP12*	19.489	5.72	5.72	9.434	1
3	102187900	A	G	rs145497598	*ZPLD1*	15.196	5.28	5.28	5.352	1
2	136107663	T	C	rs183525970	*ZRANB3*	15.459	5.42	5.42	6.253	1

Chr, chromosome; bp, base pair; Ref, reference Allele; Alt, alternate allele; SiPhy, SiPhy_29way_logOdds; phyloP100, phyloP100way_vertebrate; phastCons, phastCons100way_vertebrate.

*Genetic coordinates are given taking as a reference GRCh37/hg19, **Conservation scores produced by the different algorithms were obtained from the 2.1 version of the dbNSFP database.

**TABLE 3:** Protein prediction algorithms.

Chr	bp	Ref	Alt	rs	Gene	SIFT	Polyphen2	MutAssessor	SVM	LR	VEST3	FATHMM	LRT	MutTaster
5	56777915	C	T	rs146468598	*ACTBL2*	NA	0.461 (P)	3.37 (M)	0.7735 (D)	0.8058 (D)	0.785	−3.44 (D)	0.00001 (D)	0.999 (D)
10	81925866	T	C	rs72807973	*ANXA11*	0.174 (T)	0.005 (B)	−0.98 (N)	−0.915 (T)	0.0034 (T)	0.171	4.06 (T)	0.011694 (N)	1 (N)
2	238289767	T	C	rs112913396	*COL6A3*	0.005 (D)	1.0 (D)	2.82 (M)	−0.202 (T)	0.3558 (T)	0.932	0.75 (T)	0.000042 (D)	1 (D)
2	121748118	G	A	rs138987487	*GLI2*	0.054 (T)	1.0 (D)	1.89 (L)	−0.8456 (T)	0.1776 (T)	0.415	1.59 (T)	0.000001 (D)	1 (D)
4	39450295	G	A	rs143809363	*KLB*	0.035 (D)	0.18 (B)	0.695 (N)	−1.0599 (T)	0.0642 (T)	0.125	1.67 (T)	0.442178 (N)	0.994 (N)
1	39914279	C	G	rs376728302	*MACF1*	1 (T)	0.589 (P)	0.345 (N)	−1.0558 (T)	0.0883 (T)	0.546	0.78 (T)	0.000008 (D)	1 (D)
15	94841610	G	A	rs149237812	*MCTP2*	0.166 (T)	0.024 (B)	0.46 (N)	−0.8817 (T)	0.1776 (T)	0.161	−0.66 (T)	0.22127 (N)	0.999 (N)
1	149907574	G	A	rs202234090	*MTMR11*	0.02 (D)	0.932 (P)	2.045 (M)	0.2042 (D)	0.6049 (D)	0.568	−1.67 (D)	NA	1 (D)
5	175812257	G	A	rs35916027	*NOP16*	0 (D)	0.297 (B)	1.04 (L)	−1.0548 (T)	0.054 (T)	0.355	NA	0.000101 (N)	0.999 (N)
22	38061637	A	T	rs142028345	*PDXP*	1 (T)	0.087 (B)	−0.715 (N)	−1.0489 (T)	0.0258 (T)	0.25	1.59 (T)	NA	1 (D)
5	54721089	G	A	rs201703457	*PPAP2A*	0.077 (T)	0.263 (B)	1.905 (M)	−1.0907 (T)	0.2078 (T)	0.406	1.5 (T)	0.018731 (N)	1 (D)
9	79323853	C	T	rs200875180	*PRUNE2*	0.0 (D)	1.0 (D)	0.975 (L)	−0.8847 (T)	0.2006 (T)	0.202	0.31 (T)	0.000385 (D)	1 (D)
2	109382936	T	A	rs61748150	*RANBP2*	0.33 (T)	0.39 (B)	2.045 (M)	−1.0432 (T)	0.0603 (T)	0.16	1.56 (T)	NA	0.964 (N)
2	219528732	C	T	rs138321594	*RNF25*	0.005 (D)	0.223 (B)	0.895 (L)	−0.8015 (T)	0.1606 (T)	0.135	−0.21 (T)	0.020699 (N)	0.725 (N)
1	23743859	T	C	rs192746462	*TCEA3*	0.25 (T)	0 (B)	0.695 (N)	−1.0688 (T)	0.0598 (T)	0.379	NA	0.806532 (N)	0.992 (N)
14	24709965	G	A	rs17102311	*TINF2*	0.013 (D)	1.0 (D)	2.045 (M)	0.1966 (D)	0.7489 (D)	0.172	−3.19 (D)	0.000159 (N)	1 (D)
2	230723775	G	A	NA	*TRIP12*	0.017 (D)	0.994 (D)	0.695 (N)	−0.833 (T)	0.1914 (T)	0.684	0.8 (T)	0 (D)	1 (D)
3	102187900	A	G	rs145497598	*ZPLD1*	0.358 (T)	0.004 (B)	0.345 (N)	−0.6888 (T)	0.2761 (T)	0.5	−1.34 (T)	0 (D)	1 (D)
2	136107663	T	C	rs183525970	*ZRANB3*	0.001 (D)	1.0 (D)	3.255 (M)	1.0465 (D)	0.9121 (D)	0.857	−3.21 (D)	0 (D)	1 (D)

## Discussion

Despite their high heritability, the genetic architecture of psychotic disorders such as SCZ and BD has proven to be complex. Family-based studies, focused mainly on identifying rare inherited or rare *de novo* variants, have allowed identifying rare variants with very high penetrance, including CNVs (Malhotra and Sebat, 2012) and SNVs (Xu et al., 2011). Considering that psychosis is a complex phenotype and that the rare variants contribution is modulated by the genetic background (Nadeau, 2001; Koike et al., 2006; Kearney, 2011; Mitchell, 2011; Kerr et al., 2013) of the individuals carrying them, the more homogeneous the family and the closest the relationships included in the analysis the better.

### DUP3p26.3 and DUP16q23.3 Associated With Neurodevelopmental Disorders

The two CNVs identified in this pedigree are highly suggestive to play a significant role in the pathogenesis of psychosis. The duplication DUP3p26.3 has been extensively associated with neuropsychiatric disorders, including SCZ, autism spectrum disorder (ASD), BD, attention-deficit hyperactivity disorder (ADHD), ID, and Tourette syndrome (Kashevarova et al., 2014; Te Weehi et al., 2014; Hu et al., 2015; Huang et al., 2017; Mercati et al., 2017; Juan-Perez et al., 2018). This duplication alters the expression of the *CNTN6* gene, which encodes the protein contactin-6, also termed NB-3, a member of the contactin family of immunoglobulin domain-containing cell adhesion molecules (IgCAMs). IgCAMs are important signal molecules that mediate cell-cell adhesion and cell-extracellular matrix (ECM) interactions in multiple neural developmental processes, including neuronal migration, neurite outgrowth, axon guidance, and synaptogenesis (Sethi and Zaia, 2017). In mice, *Cntn6* is exclusively expressed in the CNS, where it is needed for proper orientation of dendrite growth in cortical pyramidal neurons (Ye et al., 2008) and synapse formation in the cerebellum (Sakurai et al., 2009). The presence of the duplication in the family may result in a decrease of the CNTN6 expression. Supporting this, a recent study comparing the CNTN6 expression in neurons generated from inducible pluripotent stem cells (iPSCs) derived from fibroblasts of an ID patient carrying a *CNTN6* duplication and two healthy controls found that neurons carrying the duplication had significantly lower expression of CNTN6 compared to wild-type neurons (Gridina et al., 2018). Although the crucial roles of CNTN6 during brain development, the penetrance of this duplication in our pedigree and others previously reported (Kashevarova et al., 2014; Hu et al., 2015) may be low since there are healthy family carriers in different generations.

The second duplication identified in the pedigree is the DUP16q23.3, also very suggestive of playing a major role in psychosis. This duplication alters the expression of *CDH13*, a gene that encodes another adhesion molecule, Cadherin-13, a calcium-dependent class of transmembrane protein that forms adherent junctions and participates in cell-cell recognition and signal transduction, among other functions. Cadherin-13 is highly expressed in the brain, where it may function as a negative regulator of neurite outgrowth and axon guidance (reviewed in Rivero et al., 2013). *CDH13* involvement in neuropsychiatric disorders is well documented, associated with the five major psychiatric disorders: ADHD (Neale et al., 2008), ASD (Sanders et al., 2011), MDD (Terracciano et al., 2010), BD (Xu et al., 2014), and SCZ (Børglum et al., 2014; Otsuka et al., 2015). Despite none of these associations have been significant at a genome-wide level, as pointed by Prata et al. (2019), in their review of GWAS studies from 2012 to 2019, *CDH13* is a highly relevant candidate for psychosis because the GWAS evidence involving this gene with psychosis comes from at least two independent samples. As observed for the DUP3p26.3, the penetrance of DUP16q23.3 in this pedigree also seems low. Both duplications need to co-segregate with other genetic factors to precipitate with psychosis, and the rare variant g.39914279 C > G of *MACF1* is a good candidate. Interestingly, all the carriers of the duplications DUP3p26.3 and DUP16q23.3 evidenced very low cognitive performance; therefore, it seems obvious that both duplications have also an impact not only in psychosis but also in other dimensions related to the disease, like cognition. In fact, other authors found that CNVs conferring risk for SCZ and autism affected cognition not only in patients but also in carrier controls (Stefansson et al., 2014). This supports our hypothesis that the combination of these CNVs with the rare variant in *MACF1* is precipitating with SCZ and SCA in the analyzed pedigree.

### Facts to Consider *MACF1* a Genetic Risk Factor for Psychosis

The function of the Microtubule-actin crosslinking factor 1 (MACF1), also known as actin crosslinking factor 7 (ACF7), is to connect the actin and microtubule cytoskeletons with the sites of cell-matrix and cell-cell adhesions (Hu et al., 2016), being an important modulator of cytoskeletal networks (Hu et al., 2016; Moffat et al., 2017). Defects in these cross-talks may have a profound impact on cell proliferation and differentiation, neuronal migration, neurite development, and axon guidance (Sanchez-Soriano et al., 2009; Goryunov et al., 2010; Ka et al., 2014, 2017; Ka and Kim, 2016; Dobyns et al., 2018). Such defects are hypothesized as a pathogenic mechanism for psychosis and several genes involved in them have been associated with SCZ or BD (Lewis et al., 2005; Rossignol, 2011; Muraki and Tanigaki, 2015; Ka et al., 2017). The role of MACF1 in cytoskeletal networks makes it an interesting candidate for psychosis (Ka et al., 2017). Besides, MACF1 also interacts with GSK3β, participating in the Wnt/β-catenin and GSK-3 signaling pathway, during the migration of pyramidal neurons or neurite differentiation (Chen et al., 2006; Ka et al., 2014; Ka and Kim, 2016). Moreover, MACF1 also interacts with DISC1 and DTNBP1 (Camargo et al., 2007; Costas et al., 2013). Interestingly, rare *de novo* variants have been previously described in BD patients (Kataoka et al., 2016; Han et al., 2019) and SCZ patients (Xu et al., 2012; Kenny et al., 2014; Wang et al., 2015) affecting this gene, which is highly intolerant to protein-altering variants. Intriguingly, both the variant identified in the family, g.39914279 C > G (p. T4642S), and other variants previously reported in SCZ patients, g.39827053 C > T (p. R2097W), and g. 39904999 C > T (p. R4033W), are located in the spectrin repeats domain of MACF1 which interacts with GSK3β and other proteins that regulate neuronal migration (Chen et al., 2006).

The rare variant identified in *GLI2* may also contribute to the psychosis phenotype. The transcription factor Gli Family Zinc Finger 2 (GLI2) is a downstream modulator of the Sonic Hedgehog (Shh) signaling pathway. Disruption of the Shh signaling has been associated with several neurodevelopmental disorders (Chen et al., 2018), including SCZ (Betcheva et al., 2013). Using GWAS, the *GLI2* gene has been associated with the manifestation of tardive dyskinesia in SCZ (Greenbaum et al., 2010).

Here we have demonstrated that by combining wide-genomic approaches it is currently feasible to disclose the genomic architecture associated with psychosis in highly penetrant and homogeneous pedigrees. Our results also show that the genomics of psychosis is highly heterogeneous, which makes sense given the involvement of rare variants in its etiology. In this sense, psychosis in the family can be explained by the presence of the variant g.39914279 C > G in *MACF1* in combination with the two rare duplications, but this variant does not explain the psychosis phenotype of their distant relative BD-12. The presence of two highly suggestive duplications, and the cognitive performance of the individuals carrying them, also suggests that the variant in *MACF1* is modulated by the genetic background of the family. Besides, our results confirm previous results suggesting the involvement of *MACF1, CNTN6*, and *CDH13* in the etiology of psychosis. More research is needed to further elucidate the role of *MACF1* in psychosis as well as its interaction with *CNTN6* and *CDH13*.

## Data Availability Statement

The raw data will be available under the accession numbers: EGAS0001004791 and EGAD00010002030 Array_data.

## Ethics Statement

The studies involving human participants were reviewed and approved by Ethics Committee of the Balearic Islands (CEI-IB). The patients/participants provided their written informed consent to participate in this study.

## Author Contributions

JP-F performed most of the analyses, including WES and CNV analysis, and wrote the manuscript. FC recruited the patients and performed their clinical characterization. LR-G, AM-D, and BB-C performed INDELS validations and digital droplet PCR. VA and DM performed SNV prioritization analyses. BO-V performed the DNA extraction and the DNA quality control. CV and CS performed Sanger sequencing validations. JL, GO, DH-S, KS, and AF contributed to NGS data analyses and experimental design. CV-B conceived and designed the analysis, supervised the study, contributed to analyze the NGS data and gene ontology, and wrote the manuscript. All authors read and approved the final manuscript.

## Conflict of Interest

The authors declare that the research was conducted in the absence of any commercial or financial relationships that could be construed as a potential conflict of interest.

## Footnotes

1 http://www.bio-rad.com/en-us/product/qx200-droplet-digital-pcr-system

2 https://www.bioinformatics.babraham.ac.uk/projects/fastqc/

3 https://cutadapt.readthedocs.io/en/stable/

4 https://github.com/lh3/bwa/releases/tag/0.7.12

5 https://github.com/samtools/samtools/releases/tag/1.1

6 https://github.com/broadinstitute/picard/releases/tag/1.118

7 https://github.com/broadinstitute/gatk/releases

8 http://snpeff.sourceforge.net/

9 http://genic-intolerance.org/

10 https://gnomad.broadinstitute.org/

11 www.1000genomes.org

12 http://software.broadinstitute.org/software/igv/

13 http://varelect.genecards.org/

14 https://www.disgenet.org

15 http://dgv.tcag.ca/dgv/app/home

16 https://decipher.sanger.ac.uk/

17 http://varsome.com

18 https://www.uniprot.org

## Supplementary Material

Supplementary Table 1 Clinical characterization. PANSS, Positive and Negative Syndrome Scale.

Supplementary Table 2 CNVs identified. Chr, Chromosome; bp, base pair; Start (bp), Starting position of the CNV; End (bp), Ending position of the CNV; Size (bp), Size of the CNV in bp; ^∗^Genetic coordinates are given taking as a reference GRCh37/hg19.

Supplementary Table 3 Validation by Digital PCR (ddPCR). CNV name, Copy Number Name; Chr, Chromosome; bp, base pair; ^∗^Genetic coordinates are given taking as a reference GRCh37/hg19; ^∗∗^Amplicon, Size of the amplicon in bp and its coordinates.

Supplementary Table 4 Cognitive assessment. TMT, Trail Making Test (Part A); BACS, Brief Assessment of Cognition in Schizophrenia (Symbol coding); HVLT-R, Hopkins Verbal Learning Test-Revised; WMS-III, Wechsler Memory Scale-Third Edition (Spatial Span); LNS, Letter-Number Span Test; NAB Mazes, Neuropsychological Assessment Battery Mazes; BVMT-R, Brief Visuospatial Memory Test-Revised; Fluency, Category Fluency Test, Animal naming; MSCEIT, Mayer-Salovey-Caruso Emotional Intelligence Test; CPT-IP, Continuous Performance Test-Identical Pairs; Pi, Percentile; Overall Composite Score: It is a cognitive index which is obtained summing all the tests scores and standardizing the result to a T score. ^∗^Low scores in comparison to reference group according to Mucci et al. (2017).

Supplementary Table 5 Residual variation intolerance score (RVIS) and loss-of-function observed/expected upper bound fraction (LOEUF). RVIS, Residual Variation Intolerance Score; LOEUF, Loss-of-function Observed/Expected Upper bound Fraction; ^∗^Percentiles are provided in parenthesis.

## References

[B1] Bateman A. (2019). UniProt: a worldwide hub of protein knowledge. *Nucleic Acids Res.* 47 D506–D515. 30395287 10.1093/nar/gky1049 PMC6323992

[B2] Bergen S. E. O’Dushlaine C. T. Ripke S. Lee P. H. Ruderfer D. M. Akterin S. (2012). Genome-wide association study in a Swedish population yields support for greater CNV and MHC involvement in schizophrenia compared with bipolar disorder. *Mol. Psychiatry* 17 880–886. 10.1038/mp.2012.73 22688191 PMC3724337

[B3] Betcheva E. T. Yosifova A. G. Mushiroda T. Kubo M. Takahashi A. Karachanak S. K. (2013). Whole-genome-wide association study in the Bulgarian population reveals HHAT as schizophrenia susceptibility gene. *Psychiatr. Genet.* 23 11–19. 10.1097/ypg.0b013e3283586343 23142968

[B4] Bipolar Disorder and Schizophrenia Working Group of the Psychiatric Genomics Consortium (2018). Genomic dissection of bipolar disorder and schizophrenia, including 28 subphenotypes. *Cell* 173 1705–1715. 29906448 10.1016/j.cell.2018.05.046 PMC6432650

[B5] Birney E. Soranzo N. (2015). The end of the start for population sequencing. *Nature* 526 52–53. 10.1038/526052a 26432243

[B6] Børglum A. D. Demontis D. Grove J. Pallesen J. Hollegaard M. V. Pedersen C. B. (2014). Genome-wide study of association and interaction with maternal cytomegalovirus infection suggests new schizophrenia loci. *Mol. Psychiatry* 19 325–333. 10.1038/mp.2013.2 23358160 PMC3932405

[B7] Camargo L. M. Collura V. Rain J.-C. Mizuguchi K. Hermjakob H. Kerrien S. (2007). Disrupted in Schizophrenia 1 interactome: evidence for the close connectivity of risk genes and a potential synaptic basis for schizophrenia. *Mol. Psychiatry* 12 74–86. 10.1038/sj.mp.4001880 17043677

[B8] Carter H. Douville C. Stenson P. D. Cooper D. N. Karchin R. (2013). Identifying Mendelian disease genes with the variant effect scoring tool. *BMC Genomics* 14(Suppl. 3):S3. 10.1186/1471-2164-14-S3-S3 23819870 PMC3665549

[B9] Chen H.-J. Lin C.-M. Lin C.-S. Perez-Olle R. Leung C. L. Liem R. K. H. (2006). The role of microtubule actin in the Wnt (MACF1) signaling pathway. *Genes Dev.* 20 1933–1945. 10.1101/gad.1411206 16815997 PMC1522081

[B10] Chen S. D. Yang J. L. Hwang W. C. Yang D. I. (2018). Emerging roles of sonic hedgehog in adult neurological diseases: neurogenesis and beyond. *Int. J. Mol. Sci.* 19:2423. 10.3390/ijms19082423 30115884 PMC6121355

[B11] Chun S. Fay J. C. (2009). Identification of deleterious mutations within three human genomes. *Genome Res.* 19 1553–1561. 10.1101/gr.092619.109 19602639 PMC2752137

[B12] Cingolani P. Platts A. Wang L. L. Coon M. Nguyen T. Wang L. (2012). A program for annotating and predicting the effects of single nucleotide polymorphisms, SnpEff: SNPs in the genome of *Drosophila melanogaster* strain w1118; iso-2; iso-3. *Fly (Austin)* 6 80–92. 10.4161/fly.19695 22728672 PMC3679285

[B13] Costas J. Suárez-Rama J. J. Carrera N. Paz E. Páramo M. Agra S. (2013). Role of DISC1 interacting proteins in schizophrenia risk from genome-wide analysis of missense SNPs. *Ann. Hum. Genet.* 77 504–512. 10.1111/ahg.12037 23909765

[B14] Davydov E. V. Goode D. L. Sirota M. Cooper S. M. Sidow A. Batzoglou S. (2010). Identifying a high fraction of the human genome to be under selective constraint using GERP++. *PLoS Comput. Biol.* 6:e1001025. 10.1371/journal.pcbi.1001025 21152010 PMC2996323

[B15] Depristo M. A. Banks E. Poplin R. Garimella K. V. Maguire J. R. Hartl C. (2011). A framework for variation discovery and genotyping using next-generation DNA sequencing data. *Nat. Genet.* 43 491–501. 21478889 10.1038/ng.806 PMC3083463

[B16] Dobyns W. B. Aldinger K. A. Ishak G. E. Mirzaa G. M. Timms A. E. Grout M. E. (2018). MACF1 mutations encoding highly conserved zinc-binding residues of the GAR domain cause defects in neuronal migration and axon guidance. *Am. J. Hum. Genet.* 103 1009–1021. 10.1016/j.ajhg.2018.10.019 30471716 PMC6288423

[B17] Dong C. Wei P. Jian X. Gibbs R. Boerwinkle E. Wang K. (2015). Comparison and integration of deleteriousness prediction methods for nonsynonymous SNVs in whole exome sequencing studies. *Hum. Mol. Genet.* 24 2125–2137. 10.1093/hmg/ddu733 25552646 PMC4375422

[B18] Felsenstein J. Churchill G. A. (1996). A hidden markov model approach to variation among sites in rate of evolution. *Mol. Biol. Evol.* 13 93–104. 10.1093/oxfordjournals.molbev.a025575 8583911

[B19] Firth H. V. Richards S. M. Bevan A. P. Clayton S. Corpas M. Rajan D. (2009). DECIPHER: database of chromosomal imbalance and phenotype in humans using ensembl resources. *Am. J. Hum. Genet.* 84 524–533. 10.1016/j.ajhg.2009.03.010 19344873 PMC2667985

[B20] Garber M. Guttman M. Clamp M. Zody M. C. Friedman N. Xie X. (2009). Identifying novel constrained elements by exploiting biased substitution patterns. *Bioinformatics* 25 54–62. 19478016 10.1093/bioinformatics/btp190 PMC2687944

[B21] GBD 2016 Disease and Injury Incidence and Prevalence Collaborators (2017). Global, regional, and national incidence, prevalence, and years lived with disability for 328 diseases and injuries for 195 countries, 1990-2016: a systematic analysis for the global burden of disease study 2016. *Lancet* 390 1211–1259. 28919117 10.1016/S0140-6736(17)32154-2 PMC5605509

[B22] Genovese G. Fromer M. Stahl E. A. Ruderfer D. M. Chambert K. Landén M. (2016). Increased burden of ultra-rare protein-altering variants among 4,877 individuals with schizophrenia. *Nat. Neurosci.* 19 1433–1441. 10.1038/nn.4402 27694994 PMC5104192

[B23] Goryunov D. He C. Z. Lin C. S. Leung C. L. Liem R. K. H. (2010). Nervous-tissue-specific elimination of microtubule-actin crosslinking factor 1a results in multiple developmental defects in the mouse brain. *Mol. Cell. Neurosci.* 44 1–14. 10.1016/j.mcn.2010.01.010 20170731 PMC2847646

[B24] Greenbaum L. Alkelai A. Rigbi A. Kohn Y. Lerer B. (2010). Evidence for association of the GLI2 gene with tardive dyskinesia in patients with chronic schizophrenia. *Mov. Disord.* 25 2809–2817. 10.1002/mds.23377 20939080

[B25] Gridina M. M. Matveeva N. M. Fishman V. S. Menzorov A. G. Kizilova H. A. Beregovoy N. A. (2018). Allele-specific biased expression of the CNTN6 gene in iPS cell-derived neurons from a patient with intellectual disability and 3p26.3 microduplication involving the CNTN6 gene. *Mol. Neurobiol.* 55 6533–6546. 10.1007/s12035-017-0851-5 29327201

[B26] Grozeva D. Conrad D. F. Barnes C. P. Hurles M. Owen M. J. O’Donovan M. C. (2012). Independent estimation of the frequency of rare CNVs in the UK population confirms their role in schizophrenia. *Schizophr. Res.* 135 1–7. 10.1016/j.schres.2011.11.004 22130109 PMC3315675

[B27] Han M. R. Han K. M. Kim A. Kang W. Kang Y. Kang J. (2019). Whole-exome sequencing identifies variants associated with structural MRI markers in patients with bipolar disorders. *J. Affect. Disord.* 249 159–168. 10.1016/j.jad.2019.02.028 30772743

[B28] Hilker R. Helenius D. Fagerlund B. Skytthe A. Christensen K. Werge T. M. (2018). Heritability of schizophrenia and schizophrenia spectrum based on the nationwide Danish twin register. *Biol. Psychiatry* 83 492–498. 10.1016/j.biopsych.2017.08.017 28987712

[B29] Hu J. Liao J. Sathanoori M. Kochmar S. Sebastian J. Yatsenko S. A. (2015). CNTN6 copy number variations in 14 patients: a possible candidate gene for neurodevelopmental and neuropsychiatric disorders. *J. Neurodev. Disord.* 7:26. 10.1186/s11689-015-9122-9 PMC4528395 26257835

[B30] Hu L. Su P. Li R. Yin C. Zhang Y. Shang P. (2016). Isoforms, structures, and functions of versatile spectraplakin MACF1. *BMB Rep.* 49 37–44. 10.5483/bmbrep.2016.49.1.185 26521939 PMC4914211

[B31] Huang A. Y. Yu D. Davis L. K. Sul J. H. Tsetsos F. Ramensky V. (2017). Rare copy number variants in NRXN1 and CNTN6 increase risk for Tourette syndrome. *Neuron* 94 1101–1111.e7. 28641109 10.1016/j.neuron.2017.06.010 PMC5568251

[B32] Juan-Perez C. Farrand S. Velakoulis D. (2018). Schizophrenia and epilepsy as a result of maternally inherited CNTN6 copy number variant. *Schizophr. Res.* 202 111–112. 10.1016/j.schres.2018.06.062 29983269

[B33] Ka M. Kim W.-Y. (2016). Microtubule-actin crosslinking factor 1 is required for dendritic arborization and axon outgrowth in the developing brain. *Mol. Neurobiol.* 53 6018–6032. 10.1007/s12035-015-9508-4 26526844 PMC4853297

[B34] Ka M. Jung E. M. Mueller U. Kim W. Y. (2014). MACF1 regulates the migration of pyramidal neurons via microtubule dynamics and GSK-3 signaling. *Dev. Biol.* 395 4–18. 10.1016/j.ydbio.2014.09.009 25224226 PMC4190130

[B35] Ka M. Moffat J. J. Kim W. Y. (2017). MACF1 controls migration and positioning of cortical GABAergic interneurons in mice. *Cereb. Cortex* 27 5525–5538. 27756764 10.1093/cercor/bhw319 PMC6075562

[B36] Karczewski K. J. Francioli L. C. Tiao G. Cummings B. B. Alföldi J. Wang Q. (2019). Variation across 141,456 human exomes and genomes reveals the spectrum of loss-of-function intolerance across human protein-coding genes. *bioRxiv* [Preprint] 10.1101/531210

[B37] Kashevarova A. A. Nazarenko L. P. Schultz-Pedersen S. Skryabin N. A. Salyukova O. A. Chechetkina N. N. (2014). Single gene microdeletions and microduplication of 3p26.3 in three unrelated families: CNTN6 as a new candidate gene for intellectual disability. *Mol. Cytogenet.* 7:97. 10.1186/s13039-014-0097-0 PMC4299808 25606055

[B38] Kataoka M. Matoba N. Sawada T. Kazuno A. A. Ishiwata M. Fujii K. (2016). Exome sequencing for bipolar disorder points to roles of de novo loss-of-function and protein-altering mutations. *Mol. Psychiatry* 21 885–893. 10.1038/mp.2016.69 27217147 PMC5414074

[B39] Kearney J. A. (2011). Genetic modifiers of neurological disease. *Curr. Opin. Genet. Dev.* 21 349–353. 10.1016/j.gde.2010.12.007 21251811 PMC3105121

[B40] Kenny E. M. Cormican P. Furlong S. Heron E. Kenny G. Fahey C. (2014). Excess of rare novel loss-of-function variants in synaptic genes in schizophrenia and autism spectrum disorders. *Mol. Psychiatry* 19 872–879. 10.1038/mp.2013.127 24126926

[B41] Kerr T. M. Muller C. L. Miah M. Jetter C. S. Pfeiffer R. Shah C. (2013). Genetic background modulates phenotypes of serotonin transporter Ala56 knock-in mice. *Mol. Autism* 4 1–11. 24083388 10.1186/2040-2392-4-35 PMC3851031

[B42] Koike H. Arguello P. A. Kvajo M. Karayiorgou M. Gogos J. A. (2006). Disc1 is mutated in the 129S6/SvEv strain and modulates working memory in mice. *Proc. Natl. Acad. Sci. U.S.A.* 103 3693–3697. 10.1073/pnas.0511189103 16484369 PMC1450143

[B43] Kopanos C. Tsiolkas V. Kouris A. Chapple C. E. Aguilera M. A. Meyer R. (2019). VarSome: the human genomic variant search engine. *Bioinformatics* 35 1978–1980. 10.1093/bioinformatics/bty897 30376034 PMC6546127

[B44] Kumar P. Henikoff S. Ng P. C. (2009). Predicting the effects of coding non-synonymous variants on protein function using the SIFT algorithm. *Nat. Protoc.* 4 1073–1082. 10.1038/nprot.2009.86 19561590

[B45] Lee S. H. DeCandia T. R. Ripke S. Yang J. Schizophrenia Psychiatric Genome-Wide Association Study Consortium (PGC-SCZ), International Schizophrenia Consortium (ISC), (2012). Estimating the proportion of variation in susceptibility to schizophrenia captured by common SNPs. *Nat. Genet.* 44 247–250. 10.1038/ng.1108 22344220 PMC3327879

[B46] Lek M. Karczewski K. J. Minikel E. V. Samocha K. E. Banks E. Fennell T. (2016). Analysis of protein-coding genetic variation in 60,706 humans. *Nature* 536 285–291. 27535533 10.1038/nature19057 PMC5018207

[B47] Lewis D. A. Hashimoto T. Volk D. W. (2005). Cortical inhibitory neurons and schizophrenia. *Nat. Rev. Neurosci.* 6 312–324. 10.1038/nrn1648 15803162

[B48] Lichtenstein P. Yip B. H. Björk C. Pawitan Y. Cannon T. D. Sullivan P. F. (2009). Common genetic determinants of schizophrenia and bipolar disorder in Swedish families: a population-based study. *Lancet* 373 234–239. 10.1016/s0140-6736(09)60072-6 19150704 PMC3879718

[B49] MacDonald J. R. Ziman R. Yuen R. K. C. Feuk L. Scherer S. W. (2014). The database of genomic variants: a curated collection of structural variation in the human genome. *Nucleic Acids Res.* 42 986–992. 10.1093/nar/gkt958 PMC3965079 24174537

[B50] Malhotra D. Sebat J. (2012). CNVs: harbingers of a rare variant revolution in psychiatric genetics. *Cell* 148 1223–1241. 10.1016/j.cell.2012.02.039 22424231 PMC3351385

[B51] Manolio T. A. Collins F. S. Cox N. J. Goldstein D. B. Hindorff L. A. Hunter D. J. (2009). Finding the missing heritability of complex diseases. *Nature* 461 747–753. 19812666 10.1038/nature08494 PMC2831613

[B52] Mercati O. Huguet G. Danckaert A. André-Leroux G. Maruani A. Bellinzoni M. (2017). CNTN6 mutations are risk factors for abnormal auditory sensory perception in autism spectrum disorders. *Mol. Psychiatry* 22 625–633. 10.1038/mp.2016.61 27166760 PMC5378808

[B53] Mitchell K. J. (2011). The genetics of neurodevelopmental disease. *Curr. Opin. Neurobiol.* 21 197–203. 20832285 10.1016/j.conb.2010.08.009

[B54] Moffat J. J. Ka M. Jung E.-M. Smith A. L. Kim W.-Y. (2017). The role of MACF1 in nervous system development and maintenance. *Semin. Cell Dev. Biol.* 69 9–17. 10.1016/j.semcdb.2017.05.020 28579452 PMC5583038

[B55] Moreno-Küstner B. Martín C. Pastor L. (2018). Prevalence of psychotic disorders and its association with methodological issues. A systematic review and meta-analyses. *PLoS One* 13:e0195687. 10.1371/journal.pone.0195687 29649252 PMC5896987

[B56] Mucci A. Galderisi S. Green M. F. Nuechterlein K. Rucci P. Gibertoni D. (2017). Familial aggregation of MATRICS consensus cognitive battery scores in a large sample of outpatients with schizophrenia and their unaffected relatives. *Psychol. Med.* 48 1359–1366. 10.1017/S0033291717002902 29017620

[B57] Muraki K. Tanigaki K. (2015). Neuronal migration abnormalities and its possible implications for schizophrenia. *Front. Neurosci.* 9:74. 10.3389/fnins.2015.00074 25805966 PMC4354421

[B58] Nadeau J. H. (2001). Modifier genes in mice and humans. *Nat. Rev. Genet.* 2 165–174. 10.1038/35056009 11256068

[B59] Neale B. M. Lasky-Su J. Anney R. Franke B. Zhou K. Maller J. B. (2008). Genome-wide association scan of attention deficit hyperactivity disorder. *Am. J. Med. Genet. Part B Neuropsychiatr. Genet.* 147 1337–1344. 10.1002/ajmg.b.30866 PMC2831205 18980221

[B60] Otsuka I. Watanabe Y. Hishimoto A. Boku S. Mouri K. Shiroiwa K. (2015). Association analysis of the Cadherin13 gene with schizophrenia in the Japanese population. *Neuropsychiatr. Dis. Treat.* 11 1381–1393. 10.2147/ndt.s84736 26082635 PMC4461090

[B61] Peiffer D. A. Le J. M. Steemers F. J. Chang W. Jenniges T. Garcia F. (2006). High-resolution genomic profiling of chromosomal aberrations using Infinium whole-genome genotyping. *Genome Res.* 16 1136–1148. 10.1101/gr.5402306 16899659 PMC1557768

[B62] Petrovski S. Wang Q. Heinzen E. L. Allen A. S. Goldstein D. B. (2013). Genic intolerance to functional variation and the interpretation of personal genomes. *PLoS Genet.* 9:e1003709. 10.1371/journal.pgen.1003709 23990802 PMC3749936

[B63] Piñero J. Ramírez-Anguita J. M. Saüch-Pitarch J. Ronzano F. Centeno E. Sanz F. (2020). The DisGeNET knowledge platform for disease genomics: 2019 update. *Nucleic Acids Res.* 48 D845–D855. 31680165 10.1093/nar/gkz1021 PMC7145631

[B64] Pollard K. S. Hubisz M. J. Rosenbloom K. R. Siepel A. (2010). Detection of nonneutral substitution rates on mammalian phylogenies. *Genome Res.* 20 110–121. 10.1101/gr.097857.109 19858363 PMC2798823

[B65] Prata D. P. Costa-Neves B. Cosme G. Vassos E. (2019). Unravelling the genetic basis of schizophrenia and bipolar disorder with GWAS: a systematic review. *J. Psychiatr. Res.* 114 178–207. 10.1016/j.jpsychires.2019.04.007 31096178

[B66] Purcell S. M. Moran J. L. Fromer M. Ruderfer D. Solovieff N. Roussos P. (2014). A polygenic burden of rare disruptive mutations in schizophrenia. *Nature* 506 185–190. 10.1038/nature12975 24463508 PMC4136494

[B67] Rees E. Walters J. T. R. Georgieva L. Isles A. R. Chambert K. D. Richards A. L. (2014). Analysis of copy number variations at 15 schizophrenia-associated loci. *Br. J. Psychiatry* 204 108–114. 10.1192/bjp.bp.113.131052 24311552 PMC3909838

[B68] Reva B. Antipin Y. Sander C. (2007). Determinants of protein function revealed by combinatorial entropy optimization. *Genome Biol.* 8:R232. 10.1186/gb-2007-8-11-r232 PMC2258190 17976239

[B69] Rivero O. Sich S. Popp S. Schmitt A. Franke B. Lesch K.-P. (2013). Impact of the ADHD-susceptibility gene CDH13 on development and function of brain networks. *Eur. Neuropsychopharmacol.* 23 492–507. 10.1016/j.euroneuro.2012.06.009 22795700

[B70] Rossignol E. (2011). Genetics and function of neocortical GABAergic interneurons in neurodevelopmental disorders. *Neural Plast.* 2011:649325. 10.1155/2011/649325 PMC3159129 21876820

[B71] Sakurai K. Toyoshima M. Ueda H. Matsubara K. Takeda Y. Karagogeos D. (2009). Contribution of the neural cell recognition molecule NB-3 to synapse formation between parallel fibers and Purkinje cells in mouse. *Dev. Neurobiol.* 69 811–824. 10.1002/dneu.20742 19672956

[B72] Sanchez-Soriano N. Travis M. Dajas-Bailador F. Gonçalves-Pimentel C. Whitmarsh A. J. Prokop A. (2009). Mouse ACF7 and *Drosophila* short stop modulate filopodia formation and microtubule organisation during neuronal growth. *J. Cell Sci.* 122 2534–2542. 10.1242/jcs.046268 19571116 PMC2704885

[B73] Sanders S. J. Ercan-Sencicek A. G. Hus V. Luo R. Murtha R. T. Moreno-De-Luca D. (2011). Multiple recurrent De Novo CNVs, including duplications of the 7q11.23 Williams syndrome region, are strongly associated with autism. *Neuron* 70 863–885. 21658581 10.1016/j.neuron.2011.05.002 PMC3939065

[B74] Schizophrenia Working Group of the Psychiatric Genomics Consortium (2014). Biological insights from 108 schizophrenia-associated genetic loci. *Nature* 511 421–427. 10.1038/nature13595 25056061 PMC4112379

[B75] Schwarz J. M. Rödelsperger C. Schuelke M. Seelow D. (2010). MutationTaster evaluates disease-causing potential of sequence alterations. *Nat. Methods* 7 575–576. 10.1038/nmeth0810-575 20676075

[B76] Sethi M. K. Zaia J. (2017). Extracellular matrix proteomics in schizophrenia and Alzheimer’s disease. *Anal. Bioanal. Chem.* 409 379–394. 10.1007/s00216-016-9900-6 27601046 PMC5203946

[B77] Shihab H. A. Gough J. Cooper D. N. Stenson P. D. Barker G. L. A. Edwards K. J. (2013). Predicting the functional, molecular, and phenotypic consequences of amino acid substitutions using Hidden Markov models. *Hum. Mutat.* 34 57–65. 10.1002/humu.22225 23033316 PMC3558800

[B78] Singh T. Kurki M. I. Curtis D. Purcell S. M. Crooks L. McRae J. (2016). Rare loss-of-function variants in SETD1A are associated with schizophrenia and developmental disorders. *Nat. Neurosci.* 19 571–577. 26974950 10.1038/nn.4267 PMC6689268

[B79] Stefansson H. Meyer-Lindenberg A. Steinberg S. Magnusdottir B. Morgen K. Arnarsdottir S. (2014). CNVs conferring risk of autism or schizophrenia affect cognition in controls. *Nature* 505 361–366. 10.1038/nature12818 24352232

[B80] Steinberg S. Gudmundsdottir S. Sveinbjornsson G. Suvisaari J. Paunio T. Torniainen-Holm M. (2017). Truncating mutations in RBM12 are associated with psychosis. *Nat. Genet.* 49 1251–1254. 10.1038/ng.3894 28628109

[B81] Stelzer G. Plaschkes I. Oz-Levi D. Alkelai A. Olender T. Zimmerman S. (2016). VarElect: the phenotype-based variation prioritizer of the GeneCards suite. *BMC Genomics* 17:444. 10.1186/s12864-016-2722-2 27357693 PMC4928145

[B82] Szatkiewicz J. P. Fromer M. Nonneman R. J. Ancalade N. Johnson J. S. Stahl E. A. (2020). Characterization of single gene copy number variants in schizophrenia. *Biol. Psychiatry* 87 736–744. 31767120 10.1016/j.biopsych.2019.09.023 PMC7103483

[B83] Te Weehi L. Maikoo R. Mc Cormack A. Mazzaschi R. Ashton F. Zhang L. (2014). Microduplication of 3p26.3 implicated in cognitive development. *Case Rep. Genet.* 2014:295359. 10.1155/2014/295359 PMC3978399 24778888

[B84] Terracciano A. Tanaka T. Sutin A. R. Sanna S. Deiana B. Lai S. (2010). Genome-wide association scan of trait depression. *Biol. Psychiatry* 68 811–817. 10.1016/j.biopsych.2010.06.030 20800221 PMC2955852

[B85] The International Schizophrenia Consortium (2008). Rare chromosomal deletions and duplications increase risk of schizophrenia. *Nature* 455 237–241. 10.1038/nature07239 18668038 PMC3912847

[B86] Thorvaldsdottir H. Robinson J. T. Mesirov J. P. (2013). Integrative genomics viewer (IGV): high-performance genomics data visualization and exploration. *Brief. Bioinform.* 14 178–192. 10.1093/bib/bbs017 22517427 PMC3603213

[B87] Wang Q. Li M. Yang Z. Hu X. Wu H. M. Ni P. (2015). Increased co-expression of genes harboring the damaging de novo mutations in Chinese schizophrenic patients during prenatal development. *Sci. Rep.* 5:18209. 10.1038/srep18209 PMC4678883 26666178

[B88] Xu B. Ionita-Laza I. Roos J. L. Boone B. Woodrick S. Sun Y. (2012). De novo gene mutations highlight patterns of genetic and neural complexity in schizophrenia. *Nat. Genet.* 44 1365–1369. 10.1038/ng.2446 23042115 PMC3556813

[B89] Xu B. Roos J. L. Dexheimer P. Boone B. Plummer B. Levy S. (2011). Exome sequencing supports a de novo mutational paradigm for schizophrenia. *Nat. Genet.* 43 864–868. 10.1038/ng.902 21822266 PMC3196550

[B90] Xu W. Cohen-Woods S. Chen Q. Noor A. Knight J. Hosang G. (2014). Genome-wide association study of bipolar disorder in Canadian and UK populations corroborates disease loci including SYNE1 and CSMD1. *BMC Med. Genet.* 15:2. 10.1186/1471-2350-15-2 24387768 PMC3901032

[B91] Ye H. Tan Y. L. J. Ponniah S. Takeda Y. Wang S.-Q. Schachner M. (2008). Neural recognition molecules CHL1 and NB-3 regulate apical dendrite orientation in the neocortex via PTPα. *EMBO J.* 27 188–200. 10.1038/sj.emboj.7601939 18046458 PMC2206121

